# A rare congenital anomaly of urinary bladder – “Bladder ears”

**DOI:** 10.4103/0971-4065.73440

**Published:** 2010-10

**Authors:** G. Lakshminarayana, A. Mathew, R. Rajesh, G. Kurien, V. N. Unni

**Affiliations:** Department of Nephrology, Amrita Institute of Medical Sciences & Research, Kochi, India

An 8-year-old girl presented with a history of a febrile illness with painless macrohematuria of 2 days duration. The hematuria was present throughout and the stream of urine was uniformly stained. There was no history of trauma or passing of stones in urine. Hematuria cleared subsequently in 3 days without any specific treatment. On examination, her weight and height were appropriate for her age; BP was 110/80mmHg, there was no edema or pallor; systemic examination was unremarkable.

On evaluation there was no proteinuria or pyuria. She had hemoglobin of 12.3 g/dL, serum creatinine of 0.6 mg/dL, and serum electrolytes were normal (sodium, potassium, calcium, and phosphorus). And 24-h urine had calcium 54.6mg, phosphorus 166.4mg, uric acid 223.3mg, and oxalate 34mg. Urine culture was sterile. Ultrasonogram of abdomen showed normal sized kidneys (Left 8.6 × 4.4 cm and right 8.7 × 4.4cm). MDCT of abdomen did not show any abnormality. Voiding cystourethrogram (VCUG) revealed symmetrical protrusions of the urinary bladder bilaterally into the pelvis [Figure 1a] a and these outpouchings are known as “bladder ears.” The “bladder ears” anomaly was more prominent while straining and [Figure 1b] and there was no vesicoureteric reflux. There was no residual contrast in the bladder in the postvoid film [Figure 2]. On DMSA scan, there was homogenous tracer distribution in both kidneys; there were no cortical photopenic defects and the percentage DMSA uptake was equal in both kidneys. We plan to do a cystoscopy if hematuria recurs.

**Figure 1a:**
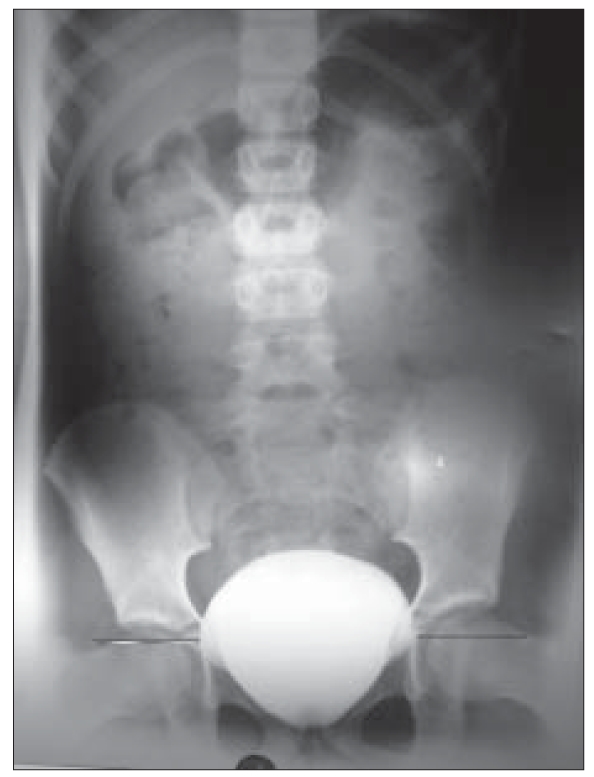
Voiding cystourethrogram (VCUG) showing symmetrical protrusions of the urinary bladder bilaterally laterally into the pelvis “Bladder ears” anomaly

**Figure 1b:**
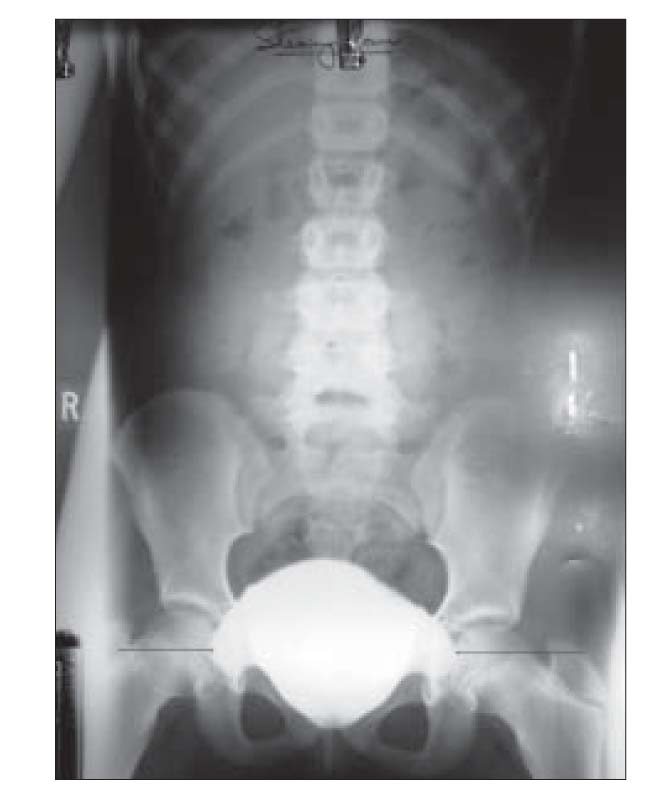
Voiding cystourethrogram (VCUG) showing symmetrical protrusions of the urinary bladder bilaterally laterally into the pelvis “Bladder ears” anomaly which appears more prominent on straining

**Figure 2:**
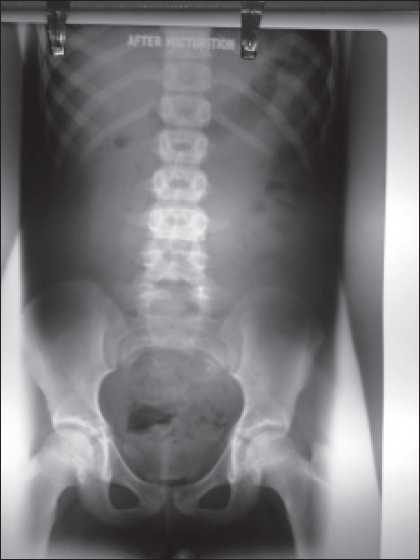
VCUG post void film showing complete emptying of bladder

After the above evaluation, she was diagnosed to have “bladder ears” anomaly. The bladder ears abnormality requires only conservative treatment and is likely to disappear with age; so she was discharged with and advice to come for follow-up if there is a further episode of hematuria or voiding symptoms.

## Discussion

Congenital anomalies of the lower urinary tract are a significant cause of morbidity in infancy and childhood. Radiological investigation is an important source of clinical information in lower urinary tract disorders. In pediatric patients with suspected underlying urologic structural anomalies, screening ultrasonography is commonly the initial diagnostic study followed by VCUG.[1–6]

Among the bladder anomalies, bladder diverticula are the commonest, and some of the uncommon ones are bladder ears, congenital hypoplasia of the bladder, bladder agenesis, duplication anomalies of the bladder, and bladder septa.[4,5,6]

Bladder diverticula are uncommon, but not rare. In a series of over 5000 children studied, the approximate incidence was 1.7% in USA.[1–3] All the other entities are uncommon or rare. Bladder anomalies are generally diagnosed in infancy or childhood. Most of these conditions have a low mortality rate and little morbidity.[1–3,5,6]

Bladder diverticula are herniations of the bladder mucosa through bladder wall musculature (detrusor muscle). Diverticular size can vary greatly, with some attaining a size equal to or greater than the volume of the bladder. Diverticula can be wide or narrow mouthed, as dictated by the size of the detrusor defect. The size of diverticular openings has functional implications because narrow-mouthed diverticula often empty poorly. Stasis of urine within diverticula can also lead to stone formation or epithelial dysplasia. Surgery is generally required when bladder diverticula cause obstruction, recurrent urinary tract infections, vesicoureteral reflux, or stone formation.[1,2,5]

Bladder ears are lateral protrusions of the bladder through the internal inguinal ring and into the inguinal canal. In infants, the bladder assumes a more abdominal position, which places it in close proximity to the internal inguinal ring. With growth, the pelvis becomes more developed, and the bladder assumes a more pelvic position. Therefore, this is rarely observed in adults. Bladder ears are observed during VCUG or intravenous pyelography (IVP), when the bladder is filled to capacity.^4^,^5^ Knowledge of this entity is important to surgeons during inguinal herniorrhaphy because there are occasional reports of partial or near total cystectomy performed under the mistaken notion that this was a large hernia sac. Surgery is unnecessary because nearly all resolve spontaneously.[6] We could not find a case report of “bladder ears anomaly” on Pubmed, Medline, and Google.

Bladder duplication and agenesis are rare and later is generally incompatible with life and both require surgical correction. Complete bladder duplication has a much higher incidence of associated anomalies necessitating surgical correction of anomalies such as fistulae between the urethra and adjacent structures. The variable anatomy of each case dictates the surgical approach. Bladder agenesis requires initial urinary diversion followed by complex urinary reconstructions, such as the creation of a continent urinary reservoir, which may be undertaken later in life.[5,6]

## References

[1] Frokiaer J, Zeidel ML, Brenner BM (2008). Brenner and Rector’s The Kidney. Urinary Tract Obstruction.

[2] Patel U (2010). Imaging and Urodynamics of the Lower Urinary Tract. Congenital Anomalies of the Bladder.

[3] Blane CE, Zerin JM, Bloom DA (1994). Bladder diverticula in children. Radiology.

[4] Berrocal T, López-Pereira P, Arjonilla A, Gutiérrez J. (2002). Anomalies of the distal ureter, bladder, and urethra in children: Embryologic, radiologic, and pathologic features. Radiographics.

[5] Sarica K, Küpeli S. (1995). Agenesis of bladder associated with multiple organ anomalies. Int Urol Nephrol.

[6] Gearhart JP (2002). Extrophy, epispadias and Bladder Anomalies. Campbell-Walsh Urology.

